# Supplementary material for: Longitudinal spin Seebeck coefficient: heat flux vs. temperature difference method
Source: Sci Rep. 2017 Apr 25;7:46752. doi: 10.1038/srep46752 (PMC5404258; doi:10.1038/srep46752)
Supplement: Supplementary Material [file srep46752-s1.pdf]

Supplementary material

Longitudinal spin Seebeck coefficient: heat flux vs. temperature difference method

A. Sola*, P. Bougiatioti, M. Kuepferling, D. Meier, G. Reiss, M. Pasquale, T. Kuschel, and V. Basso

*Istituto Nazionale di Ricerca Metrologica, Strada delle Cacce 91, 10135, Turin, Italy

a.sola@inrim.it

Ohmic contacts on the LSSE device

In order to investigate the reproducibility of the thermal measurement for the determination of the LSSE coefficient, it is necessary to have a robust reproducibility of the electrical part of the LSSE characterization. This condition is easier to achieve compared with the thermal measurement, but there is still a slight dependence on the shape of the ohmic contact. In particular, when the electrical wires are directly connected to the Pt film, the characteristic of the sample may depend on the type of contact.

In order to eliminate this dependency, we prepared the sample with two electrodes that are two 100 nm thick gold strips of the same shape at the edges of the Pt surface. The presence of these electrodes allow a reproducible detection of the LSSE driven voltage for any kind of the ohmic contacts above the electrode i.e. droplets of silver paint of different sizes or wire-bonding as reported in Figure S1.

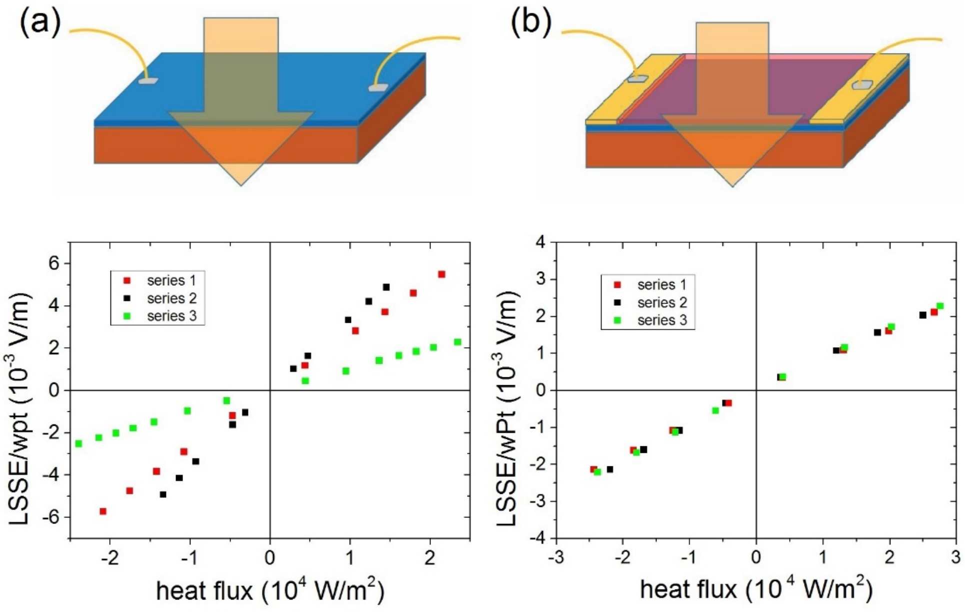

Figure S1. LSSE driven electric field as a function of the heat flux through the sample. Data obtained from direct ohmic contacts on the Pt film (a) and from the sample prepared with electrodes (b).

Figure S1 shows three series of LSSE characteristics as function of the heat flux for two different configuration of ohmic contacts. For each series we removed and subsequently restored the electrical contact. In Figure S1(a) the ohmic contact is formed directly on the Pt film, while data in Figure S1(b) refer to the presence of the gold electrodes. It is clear that the presence of the electrodes improves the reproducibility of the electric measurement for LSSE characterization.
